# Genetic Testing Contributes to Diagnosis in Cerebral Palsy: Aicardi-Goutières Syndrome as an Example

**DOI:** 10.3389/fneur.2021.617813

**Published:** 2021-04-22

**Authors:** Diane Beysen, Chania De Cordt, Charlotte Dielman, Benson Ogunjimi, Julie Dandelooy, Edwin Reyniers, Katrien Janssens, Marije M.E. Meuwissen

**Affiliations:** ^1^Department of Pediatric Neurology, Antwerp University Hospital, Edegem, Belgium; ^2^Department of Pediatrics, Antwerp University Hospital, Edegem, Belgium; ^3^Department of Pediatric Neurology, Ziekenhuis Netwerk Antwerpen Queen Paola Children's Hospital, Wilrijk, Belgium; ^4^Center for Health Economics Research & Modeling Infectious Diseases (CHERMID), Vaccine & Infectious Disease Institute (VAXINFECTIO), University of Antwerp, Antwerp, Belgium; ^5^Department of Pediatrics, Ziekenhuis Netwerk Antwerpen Paola Children's Hospital, Wilrijk, Belgium; ^6^Department of Dermatology, Antwerp University Hospital, Edegem, Belgium; ^7^Center for Medical Genetics, Antwerp University Hospital, Edegem, Belgium; ^8^Center for Medical Genetics, University of Antwerp, Wilrijk, Belgium

**Keywords:** cerebral palsy, aicardi goutières syndrome, next generation sequencing, genetic testing, genetic diagnosis

## Abstract

Cerebral palsy (CP) is a non-progressive neurodevelopmental disorder characterized by motor impairments, often accompanied by co-morbidities such as intellectual disability, epilepsy, visual and hearing impairment and speech and language deficits. Despite the established role of hypoxic–ischemic injury in some CP cases, several studies suggest that birth asphyxia is actually an uncommon cause, accounting for <10% of CP cases. For children with CP in the absence of traditional risk factors, a genetic basis to their condition is increasingly suspected. Several recent studies indeed confirm copy number variants and single gene mutations with large genetic heterogeneity as an etiology in children with CP. Here, we report three patients with spastic cerebral palsy and a genetically confirmed diagnosis of Aicardi-Goutières syndrome (AGS), with highly variable phenotypes ranging from clinically suggestive to non-specific symptomatology. Our findings suggest that AGS may be a rather common cause of CP, that frequently remains undiagnosed without additional genetic testing, as in only one case a clinical suspicion of AGS was raised. Our data show that a diagnosis of AGS must be considered in cases with spastic CP, even in the absence of characteristic brain abnormalities. Importantly, a genetic diagnosis of AGS may have significant therapeutic consequences, as targeted therapies are being developed for type 1 interferonopathies, the group of diseases to which AGS belongs. Our findings demonstrate the importance of next generation sequencing in CP patients without an identifiable cause, since targeted diagnostic testing is hampered by the often non-specific presentation.

## Introduction

Aicardi-Goutières syndrome (AGS) is a rare hereditary inflammatory disorder which usually presents in early infancy with systemic and central nervous manifestations caused by the inappropriate induction of a type I interferon-mediated immune response (1). To date, mutations in seven genes have been identified to cause AGS, namely *TREX1, RNASEH2A, RNASEH2B, RNASEH2C, SAMHD1, ADAR1*, and *IFIH1*. AGS is part of the type 1 interferonopathies. The type 1 interferon-mediated antiviral response is usually triggered by viral infections (2). In case of Aicardi-Goutières syndrome, it is hypothesized that self-nucleic acids are no longer recognized as such, leading to an inappropriate immune response with an overactive type 1 interferon signaling, even in the absence of a viral infection. Suggestive features include cerebrospinal fluid (CSF) lymphocytosis and the neuroradiological findings of calcifications of the basal ganglia and leukoencephalopathy. Neurological manifestations include progressive microcephaly, spasticity, epilepsy and developmental delay, while systemic manifestations include hepatosplenomegaly, skin rash and chilblain lesions, glaucoma and systemic lupus erythematosus-like manifestations. Although the majority of patients present with this “classical” phenotype, several studies report a broader spectrum of disease presentation, progression and outcome, with even static or slowly progressive forms presenting as cerebral palsy (CP) (3–5). Due to this clinical heterogeneity, the actual prevalence of AGS is not known.

Cerebral palsy (CP) is a clinical descriptive term that defines a heterogeneous group of non-progressive neurodevelopmental disorders of motor impairment, which co-occur with a wide range of medical conditions such as intellectual disability (ID), speech and language deficits, autism, epilepsy and visual and/or hearing impairment. CP is caused by a cerebral anomaly/dysfunction that develops during pregnancy, birth or infancy (before the age of 2 years). With a prevalence of 1.5–2.5 per 1,000 live births in high-income countries and an estimated prevalence of up to 3.6 per 1,000 children in low-income countries (6), CP is the most common cause of physical disability in childhood (7) with an important impact on the quality of life of patients and on society. Historically, inadequate oxygen delivery to the brain during birth was thought to be the leading cause of CP, but large population-based controlled studies have shown birth asphyxia to be a rather uncommon cause accounting for <10% of CP cases (8). This recent insight has led to an increased interest in genetic studies to elucidate its additional causes. However, compared to other neurodevelopmental disorders, i.e., ID and autism, large genetic studies in CP are still underrepresented (2).

Triggered by these findings, we started to perform genetic testing in our cohort of patients with a clinical diagnosis of CP in order to identify underlying molecular diagnosis. Testing involves SNP microarray to exclude pathogenic copy number variants followed by whole exome sequencing with *in silico* filtering of variants in around 200 genes associated with CP or “CP-mimics.” The latter is defined as a neurodevelopmental disorder that initially presents with a CP phenotype, but with a differing natural history and prognosis (9, 10). A full list of the genes included in our CP panel with their associated disorder, inheritance pattern and clinical characteristics is available in the supplements (Supplementary Table 1). While our study is ongoing, we already present three cases from our CP cohort with a genetically confirmed diagnosis of Aicardi-Goutières syndrome (AGS); phenotypes ranged from clearly suggestive for the disorder to non-specific findings like spastic diplegia/quadriplegia, developmental delay and non-pathognomonic brain MRI findings.

With these three cases, we want to illustrate the added value of genetic testing in CP, as this may lead to unexpected diagnoses that may alter treatment options. Moreover, a molecular diagnosis in CP patients aids in the counseling of patients and their families, as it helps them understand the cause of the condition and improves the testing possibilities in future family planning.

## Methods

In all three cases, Single Nucleotide Polymorphism (SNP) array was performed using an Illumina HumanCytoSNP-12 v2.1 beadchip on an iScan system, following manufacturer's protocols. CNV analysis was done using GenomeStudio software (Illumina) and CNV WebStore, version 2.0 as previously described (11).

Whole exome sequencing (WES) of DNA obtained from blood lymphocytes of the index case and both parents was performed on an Illumina HiSeq4000 instrument after enrichment with the Agilent SureSelect Human All Exon V5 kit. Data analysis was performed using an in-house developed pipeline following GATK Best Practice Guidelines, followed by variant annotation and filtering using VariantDB (12). We analyzed a gene panel consisting of 200 genes associated with CP and CP mimics.

In one case (case 1), sequencing of the coding exons and exon/intron boundaries of *TREX1* (NM_007248.5), *RNASEH2A/B*/*C* (NM_006397.2, NM_001142279.2, NM_032193.4), *ADAR1* (NM_001193495.2)*, SAMHD1* (NM_001363729.2) and *IFIH1* (NM_022168.4) was performed using a targeted next-generation sequencing panel for AGS (by courtesy of Yanick Crow, Laboratory of Neurogenetics and Neuroinflammation, Paris Descartes University, Sorbonne-Paris-Cité, INSERM UMR 1163, Institut Imagine, Paris, France). In addition, Multiplex Amplicon Quantification (MAQ) analysis of exon 5 of *SAMHD1* was performed.

## Case Reports

Our first case is a girl referred because of delayed motor development and gait difficulties at the age of 2 years. She is the first child of healthy, non-consanguineous parents of Caucasian origin with a normal family history. Pregnancy and birth were uneventful. Neurological examination at the time of referral demonstrated cerebral palsy with spastic diparesis; brain MRI showed non-specific, periventricular leukomalacia.

**Figure 1:**
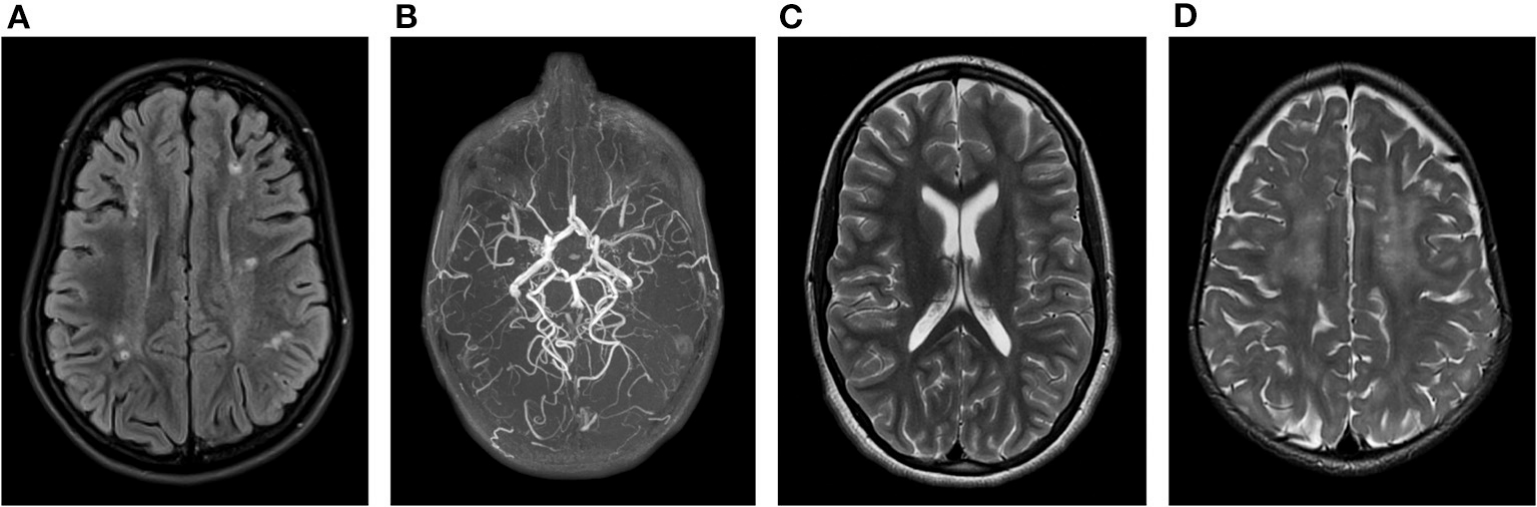
Brain MRI findings in our AGS patients: **(A)** Periventricular and frontoparietal lesions suggestive of cortical and white matter infarctions associated with **(B)** moyamoya disease at the distal left internal carotid artery and the proximal right middle cerebral artery on MR angiography in patient 1. **(C)** Isolated posterior thinning of the corpus callosum in patient 2. **(D)** Bilateral frontal periventricular microcysts and diffuse white matter hyperintensities in patient 3.

At the age of 8 years, she was diagnosed with attention deficit hyperactivity disorder and developed skin lesions on hands and toes during cold months, most likely chilblain lesions. This was supported by analysis of a skin biopsy, demonstrating a perivascular inflammation and immunofluorescence pattern suggestive for the presence of a lupus band. Brain Magnetic Resonance Imaging (MRI) at the age of 10 years demonstrated periventricular and frontoparietal white matter abnormalities suggestive of sequels of cortical and white matter infarctions and limited parietal cortical atrophy (Figure 1A). MR angiography displayed images compatible with moyamoya disease at the distal left internal carotid artery and the proximal right middle cerebral artery (Figure 1B). Ophthalmological investigations were normal. RNA analysis of blood showed an increase of interferon-related gene expression, compatible with a diagnosis of AGS. Blood analysis also showed persistently mildly increased aspartate aminotransferase (AST)/alanine aminotransferase (ALT) as well as presence of antinuclear antibodies (anti-DFS70) with normal complement.

The chilblain lesions were initially treated with a combination of a calcium antagonist and hydroxychloroquine, with little improvement. After the molecular confirmation of the AGS diagnosis, a switch to a Janus Kinase (JAK) inhibitor demonstrated good results, with normalization of AST/ALT and reduction of chilblains.

The second case is a girl referred at the age of 9 years because of developmental delay that was only noted after an episode of fever of unknown origin at the age of 10 months. The girl is the third child of healthy parents of North-African origin, who are first-degree cousins. The mother had two early miscarriages. No other relatives presented with neurological problems.

**Figure 2:**
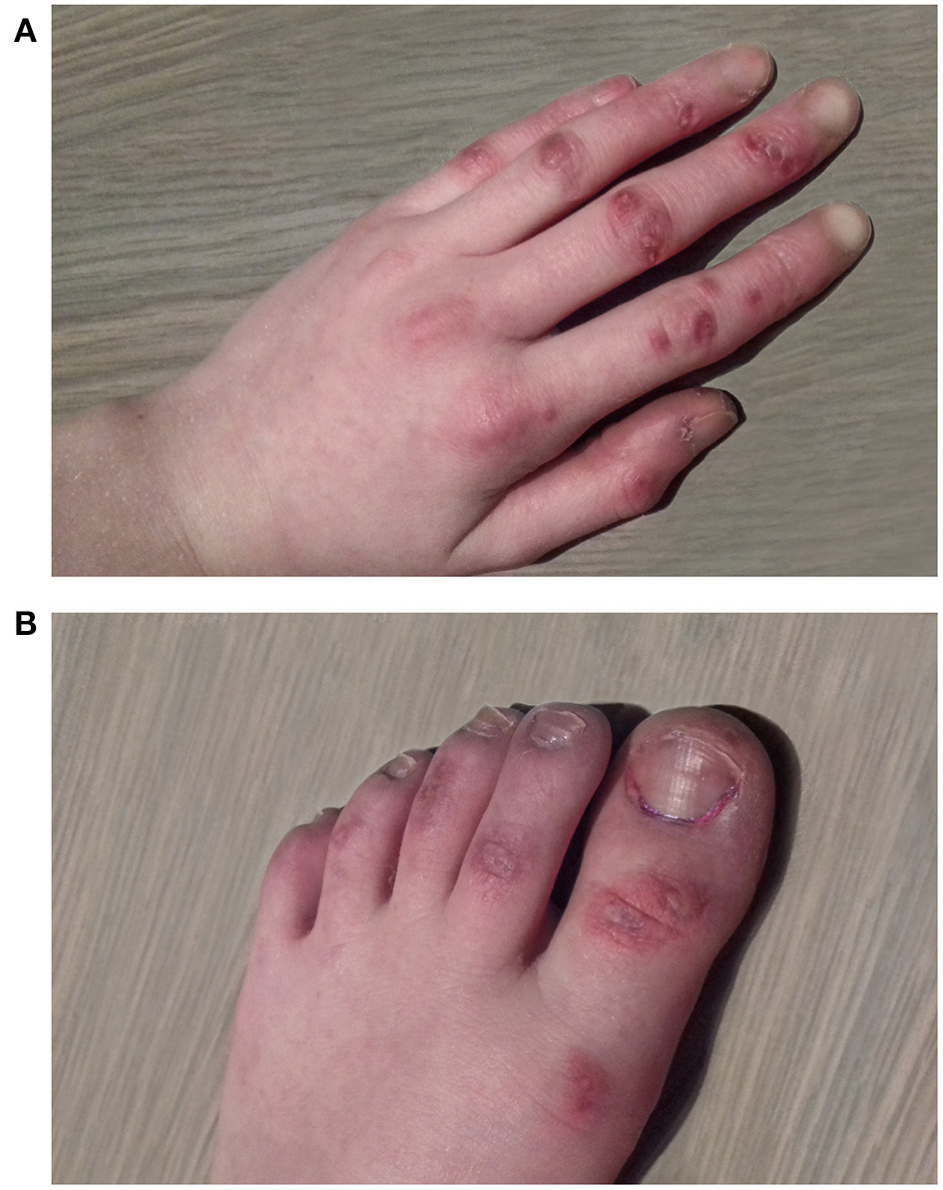
Chilblain lesions appearing in winter time on hands **(A)** and feet **(B)** of patient 2.

At initial presentation, a quadriplegia of mixed spastic and dyskinetic type was noted, more pronounced on the right side of her body. Mild intellectual disability and speech deficit were observed. During winter, she developed skin lesions on her feet which were initially attributed to pressure lesions from splints, but were later classified as chilblain lesions (Figure 2).

MRI of the brain showed posterior thinning of the corpus callosum without other abnormalities (Figure 1C). Ophthalmological evaluation was normal. At the age of 9 years, a bilateral femur-osteotomia was performed because of recurrent bilaterally occurring hip luxations. At the age of 13 years, she underwent a combined hamstring release with open hip reduction and pelvic girdle osteotomia.

Currently, at the age of 15, she can roll over independently and sit with support. Speech is limited to single words and short sentences. She developed a scoliosis and joint contractures, treated with a corset, wrist splints and baclofen. Chilblain lesions are treated with a topical corticosteroid cream with good response.

The third case is a girl referred at the age of 1½ years because of developmental delay. She is the fourth child of healthy, apparently unrelated parents of Moroccan descent. Pregnancy and birth were uneventful. At first referral, a spastic diparesis with hypertonia of both legs was noted. She had recurrent fever episodes, and parents noted a temporary relapse during these episodes, with a decrease in babbling and an increase in feeding difficulties that later resolved. At the age of 1 year, she had one episode suggestive of an epileptic seizure, but a subsequent electroencephalogram was normal. Brain MRI identified bilateral frontal periventricular cysts (Figure 1D). Metabolic investigations showed elevated lactic acid and hyperammonaemia, that both had normalized at a second evaluation. Because of the findings on brain MRI and the elevated lactic acid, genetic testing for mitochondrial disorders, both on mitochondrial and nuclear DNA, was performed, but turned out normal. Ophthalmological evaluation was normal. Currently, at the age of 2 years, she starts to sit without support and is able to take little steps with support of both hands. Speech is limited to some words. Neurological examination confirmed a spastic diplegic cerebral palsy with hyperreflexia. No chilblain lesions are noticed.

The clinical findings of our cases are summarized in Table 1.

**Table 1:** Summary of genetic and clinical findings of our AGS cases.

	**Case 1**	**Case 2**	**Case 3**
Affected gene	*SAMHD1*	*RNASEH2B*	*RNASEH2B*
Pathogenic variant	c.109G>T (p.Glu37^*^)(HET)/deletion exon 5 (HET)	c.529G>A (p.Ala177Thr)(HOM)	c.529G>A (p.Ala177Thr)(HOM)
Sex	F	F	F
History of consanguinity	–	+	–
Age at last examination	12 y, 1 m	15 y, 8 m	3 y, 6 m
Age at presentation	1 y, 10 m	8 y, 8 m	2 y, 1 m
Age at diagnosis	10 y, 3 m	14 y, 9 m	3 y, 2 m
Motor delay	+	+	+
Speech delay	+	+	–
Intellectual disability	+	+	+
Seizures	–	–	?
Chilblain lesions	+	+	–
Head circumference (cm)	46.5 (p20)	52 (p50)	48.3 (p50)
Neurological findings	Spastic diplegia ADHD	Spastic and dyskinetic quadriplegia	Spastic diplegia
Ophthalmological findings	–	–	–
Other findings	Eczema	–	–
Brain MRI	Bilateral white matter abnormalities, parietal cortical atrophy and signs of Moyamoya disease	Posterior thinning corpus callosum	Periventricular microcysts and white matter abnormalities

*HET, heterozygous; HOM, homozygous; F, female; y, years; m, months*.

## Results

SNP array did not reveal clinically relevant copy number variants in any of the cases.

In the first case, analysis of a panel for AGS showed compound heterozygosity for two pathogenic variants in the *SAMHD1* gene. Initially, only a paternally inherited c.109G>T (p.Glu37^*^) variant was identified and confirmed by Sanger sequencing. Further interrogation of the read depth suggested a possible deletion of exon 5 of the *SAMHD1*-gene, that was confirmed by MAQ analysis and was maternally inherited. Both variants are absent from GnomAD (https://gnomad.broadinstitute.org/). Truncating variants and intragenic *SAMHD1* exon deletions have previously been described as pathogenic (13–15).

In cases 2 and 3, WES demonstrated the same homozygous c.529G>A (p.Ala177Thr) variant in the a*RNASEH2B* gene. Heterozygous carriership for the mutation was confirmed in the parents. This variant was already reported as pathogenic in the literature (16, 17).

## Discussion

We describe three patients with a clinical diagnosis of spastic cerebral palsy in whom genetic testing contributed to a molecular diagnosis of Aicardi-Goutières syndrome. Based on the clinical findings in our cases, an AGS diagnosis was suspected in only one (case 1). The other two cases were identified by WES, a technology that has only recently been implemented in our routine diagnostics for CP patients. The identification of two AGS cases in approximately 100 CP cases that have been analyzed by WES at our genetic center to date suggests that AGS may be a rather common cause of CP that can remain undiagnosed without thorough genetic testing, although we acknowledge that the small sample size of our CP cohort can cause a bias in the interpretation of these data.

Our first case presented with cerebrovascular abnormalities with cortical and white matter infarctions associated with moyamoya disease. The latter is an important complication specific for *SAMHD1*-related AGS and is not seen in the other subtypes (18). Consequently, we were not surprised that a targeted AGS panel consisting of the seven genes currently known to be associated with AGS demonstrated compound heterozygosity for a *SAMHD1* non-sense mutation and a single exon deletion.

The homozygous c.529G>A (p.Ala177Thr) variant in *RNASEH2B* identified in the second and third case is a recurrent pathogenic mutation that was already reported in several cases of AGS that presented with a more non-specific phenotype, including two adult-onset cases with cerebral calcifications and chilblains, one case with chilblain lesions only and three cases presenting with spastic di/quadriplegia and preserved intellect. This, together with the non-specific phenotype of spastic CP in our two cases, may suggest a potential genotype-phenotype correlation with a tendency toward non-specific clinical features. However, a classic infantile AGS presentation was also present in two cases (16, 17).

The highly variable and sometimes rather non-specific phenotype of AGS hampers a swift diagnosis and probably results in underdiagnosis of the condition. A recent study of interferon-related gene expression in seven patients with sine causa spastic-dystonic tetraparesis led to the diagnosis of AGS in one patient by the demonstration of a “type 1 interferon signature,” a specific expression pattern of IFN-related genes identified in type 1-interferonopathies (19, 20). While an analysis of interferon-related gene expression may indeed be useful, we suggest performing genetic testing by whole exome sequencing as a first-tier strategy in CP diagnostics due to the wide differential diagnostic considerations in CP. Determining the type 1 interferon signature may be of added value in case of the identification of variants of unknown significance, or in cases highly suggestive of AGS but in which genetic testing fails to identify a pathogenic variant in one of the currently known AGS genes. One drawback of WES is that certain variant types, i.e., CNVs, or potentially relevant intronic/promoter/regulatory variants can remain undetected, rendering it possible that a genetic diagnosis is missed, while the interferon signature in these cases can still point to the diagnosis of AGS.

The importance of a genetic diagnosis of AGS cannot be overestimated: it aids in the counseling of patients and their families, offers opportunities for family planning and has therapeutic consequences, as targeted therapies for type 1 interferonopathies are emerging. Several treatment strategies modulating components of the transducing pathway of the interferon response are under study, including reverse transcriptase inhibitors (RTIs) and Janus kinase (JAK) inhibition, although further testing is needed to prove their efficacy and study their long-term effects (21, 22).

Although this report focuses on AGS patients, we want to stress the importance of genetic testing in all CP patients, since a definite diagnosis in this cohort can easily be missed solely based on the often non-specific clinical presentations.

As previously stated, large genetic studies in CP are only just emerging, but they all point to an important contribution of genetic causes (8). The first publication on the benefit of early (genetic) diagnosis dates from 2014; Leach et al. summarized several inborn errors of metabolism that can present as CP mimics, of which several are amendable to treatment (9). 1 year later, clinically relevant copy number variants (CNVs) were demonstrated in 9.6% (11/115) of CP cases (23). An additional study by Zarrei et al. in 2018 demonstrated a frequent occurrence of *de novo* CNVs in the hemiplegic CP subtype (7.2%) compared to controls (1%) (24). Also in 2015, the first WES study in a cohort of 201 CP cases demonstrated an important contribution of single nucleotide variants (SNVs), with a diagnostic yield of 14% (25). Another study in 2018 even reached a diagnostic yield of 65% using WES in 50 individuals with atypical CP; however, this study also included promising candidate genes whose role in CP has not been validated (26). A study in 2019, using a gene panel of 112 genes consisting of a combination of known disease genes and candidate genes classified as intolerant to variation, found genetic variants of possible clinical relevance in 10% of the CP cohort (27). While the genetic causes are heterogeneous, this study identified six recurrent genes that contributed to at least 4% of disease burden in CP: *COL4A1, TUBA1A, AGAP1, L1CAM, MAOB* and *KIF1A* (27).

Based on these findings, the Center of Medical Genetics in Antwerp started routinely offering SNP-array followed by trio-bases WES with analysis of a targeted gene panel consisting of around 200 genes associated with CP or CP mimics (Supplementary Table 1) in all CP cases without evident traditional risk factors as of 2019. The full results of the genetic findings in our CP cohort will be published in an additional manuscript. Due to the continuous increase in genes associated with a CP phenotype, the drawback of our approach using a selected gene panel is that pathogenic variants in genes only recently associated with CP or genes associated with neurodevelopmental disorders in which CP is only a rare presentation can be missed. A phenotype-driven genome-wide analysis may therefore be offered in the absence of pathogenic CNVs or pathogenic variant in the targeted panel. An advantage of using a targeted gene panel is that the chance of incidental findings is low.

The genetic heterogeneity associated with CP has triggered a discussion regarding the validity of CP as a clinical diagnosis. This topic was recently addressed by MacLennon et al., stating that CP is a neurodevelopmental disorder diagnosed based on clinical signs, not etiology (28). The authors present several important arguments to keep CP as a clinical diagnosis instead of replacing it with the molecularly identified disorder, including social and financial support systems that are present for CP patients, access to services, utility and accuracy of CP registries and community understanding of the term CP (28). We fully agree that the diagnosis of CP should not be altered, but it should warrant additional genetic testing to enable genetic subclassifications of CP, for example “CP caused by a pathogenic *COL4A1* variant.” Subclassifying CP based on genetic causes improves genetic counseling of families e.g., regarding future family planning and contributes to an improved and overall more personalized treatment and follow-up of CP patients.

## Data Availability Statement

The original contributions presented in the study are publicly available. This data can be found here: https://www.ncbi.nlm.nih.gov/clinvar/, accession numbers SUB9038630: SCV001478409, SUB9149334: SCV001481865.

## Ethics Statement

Ethical review and approval was not required for the study on human participants in accordance with the local legislation and institutional requirements. Written informed consent to participate in this study was provided by the participants' legal guardian/next of kin. Written informed consent was obtained from the minor(s)' legal guardian/next of kin for the publication of any potentially identifiable images or data included in this article.

## Author Contributions

This work was realized with the collaboration of all authors. DB contributed by designing and conceptualizing the study and drafting of the manuscript for intellectual content. CDC contributed by designing and conceptualizing the study and drafting of the manuscript for intellectual content. CD contributed by playing a major role in the acquisition of clinical data. BO contributed by playing a major role in the acquisition of clinical data and in the revision of the manuscript for intellectual content. JD contributed by playing a major role in the acquisition of clinical data. ER contributed by the generation and interpretation of the genomic data. KJ contributed by the interpretation of the data and by revision of the manuscript for intellectual content. MM designed and conceptualized the study, analyzed and interpreted the data and drafted the manuscript for intellectual contentment. All authors contributed to the article and approved the submitted version.

## Conflict of Interest

The authors declare that the research was conducted in the absence of any commercial or financial relationships that could be construed as a potential conflict of interest.
