# Supplementary material for: Genetic Testing Contributes to Diagnosis in Cerebral Palsy: Aicardi-Goutières Syndrome as an Example
Source: Front Neurol. 2021 Apr 22;12:617813. doi: 10.3389/fneur.2021.617813 (PMC8100223; doi:10.3389/fneur.2021.617813)
Supplement: Supplementary file 1 [file Data_Sheet_1.PDF]

GENE	CONDITION	OMIM (CONDITION)	INHERITANCE	NEUROLOGICAL FEATURES	BRAIN MRI	ADDITIONAL FEATURES	REFERENCE (IN CASE OF ABSENT OMIM NUMBER)
AADC	Aromatic L-amino acid decarboxylase (AADC) deficiency	608643	AR	Dystonia, severe neurological dysfunction, oculogyric crisis, infantile/ childhood onset			
ACOX1	Peroxisomal acyl-CoA oxidase deficiency	264470	AR	Neonatal hypotonia, seizures, dystonia, progressive neurological dysfunction	White matter hyperintensities demyelination, leukodystrophy		
ADA2	Sneddon syndrome	182410	AR	Non-inflammatory arteriopathy, cerebrovascular accidents, hemorrhage		Livedo reticularis, SLE, polyarteriitis nodosa	
ADCK3	Primary coenzyme Q10 deficiency-4/ spinocerebellar ataxia type 9	606980	AR	Cerebellar ataxia, stroke-like episodes, exercise intolerance, seizures, mild ID			
ADCY5	Familial dyskinesia with facial myokymia	600293	AD	Dystonia, choreatic movements of limbs and facial muscles, choreoform CP			
ADD3	Spastic quadriplegic cerebral palsy-3	617008	AR	Spastic quadriplegia, seizures, ID, infantile onset	White matter hyperintensities		
AFG3L2	early-onset spinocerebellar ataxia with spasticity and myoclonic epilepsy	614487	AR	spinocerebellar ataxia with spasticity and myoclonic epilepsy			
AGAP1	NA	NA	AD (DN)	Cerebral palsy			PMID: 31700678
AHI1	Joubert syndrome-3	608629	AR	Hypotonia, ataxia, spasticity	molar tooth sign, cerebellar vermis hypoplasia, polymicrogyria, corpus callosum malformations		
AIMP1	Leukodystrophy, hypomyelinating, 3	260600	AR	global developmental delay, lack of development, lack of speech acquisition, and peripheral spasticity	absence of myelination		
AKT3	Megalencephaly-polymicrogyria-polydactyly-hydrocephalus syndrome-2	615937	AD (DN)	Megalencephaly, seizures, ID	Hydrocephalus, polymicrogyria	Polydactyly, capillary malformations	
ALDH3A2	Sjogren-Larsson syndrome	270200	AR	ID, spastic paraparesis	Leukoencephalopathy	Ichthyosis, macular dystrophy	
ALDH5A1	Succinic semialdehyde dehydrogenase (SSADH) deficiency	271980	AR	developmental delay, hypotonia, mental retardation, ataxia, seizures, hyperkinetic behavior, aggression, and sleep disturbances	increased T2-weighted signals in the globus pallidi		
ALS2	Spastic paralysis, infantile onset ascending	607225	AR	ID, spastic diplegia, facial muscle weakness, amyotrophy	White matter hyperintensities, atrophy of motor cortex	Ocular movement disorders, bowel/ urinary incontinence	
ALT1	Spastic paraplegia 3A, autosomal dominant	182600	AD	Early onset spasticity			
AMPD2	Pontocerebellar hypoplasia, type 9	615809	AR	ID, spasticity, dystonia, microcephaly, seizures	cerebellar hypoplasia/ atrophy		
AP4B1	Autosomal recessive spastic paraplegia-47	614066	AR	neonatal hypotonia that progresses to hypertonia and spasticity and	Thin corpus callosum, periventricular white matter		

				severe mental retardation with poor or absent speech development	abnormalities, white matter loss, ventriculomegaly	
AP4E1	Autosomal recessive spastic paraplegia-51	613744	AR	neonatal hypotonia that progresses to hypertonia and spasticity and severe mental retardation with poor or absent speech development	Cortical atrophy, cerebellar atrophy, enlarged ventricles, diffuse white matter loss	
AP4M1	Autosomal recessive spastic paraplegia-50	612936	AR	neonatal hypotonia that progresses to hypertonia and spasticity and severe mental retardation with poor or absent speech development	Cerebellar atrophy, thin corpus callosum, ventriculomegaly, white matter lesions, decreased myelin, gliosis	
AP4S1	Autosomal recessive spastic paraplegia-52	614067	AR	neurodevelopmental disorder characterized by neonatal hypotonia that progresses to hypertonia and spasticity and severe mental retardation with poor or absent speech development	Thin or absent corpus callosum	
ARG1	Argininemia	207800	AR	spastic paraplegia, epileptic seizures, and severe mental retardation, microcephaly		
ARSA	Metachromatic leukodystrophy	250100	AR	motor symptoms, rigidity, mental deterioration, and sometimes convulsions	Demyelination	
ARX	Epileptic encephalopathy, early infantile, 1	308350	XLR	infantile spasms with severe dyskinetic quadriparesis		
ASPA	Canavan disease	271900	AR	onset in early infancy, atonia of neck muscles, hypotonia, hyperextension of legs and flexion of arms, blindness, severe mental defect, megalocephaly	Demyelination and leukodystrophy	
ATAD3A	Harel-Yoon syndrome	618810	AD	developmental delay, hypotonia, spasticity, optic atrophy, axonal neuropathy, dyskinetic CP	Cerebellar atrophy	Hypertrophic cardiomyopathy
ATL1	Autosomal dominant spastic paraplegia-3A	182600	AD	early onset spastic paraplegia		
ATM	Ataxia-telangiectasia	208900	AR	cerebellar ataxia, progressive neurologic degeneration	Cerebellar cortical degeneration	Telangiectases, immune defects, and a predisposition to malignancy
ATP7B	Wilson disease	277900	AR	Tremor, dysarthria, dystonia, dementia	abnormalities of the basal ganglia in generalized cerebral atrophy, they also noted subtle white matter abnormalities	Kayser-Fleischer rings, liver cirrhosis, hepatomegaly
AUTS2	Autosomal dominant mental retardation-26	615834	AD (DN)	intellectual disability, autism, microcephaly, cerebral palsy,		Short stature, dysmorphisms

BCKDHA	Maple syrup urine disease	248600	AR	progressive neurologic signs of altering hypotonia and hypertonia, reflecting a severe encephalopathy. Seizures and coma usually occur, followed by death if untreated	White matter signal abnormalities, diffusion abnormalities		
BCKDHB	Maple syrup urine disease	248600	AR	progressive neurologic signs of altering hypotonia and hypertonia, reflecting a severe encephalopathy. Seizures and coma usually occur, followed by death if untreated	White matter signal abnormalities, diffusion abnormalities		
BSCL2	Silver spastic paraplegia syndrome	270685	AD	spastic paraparesis and severe amyotrophy of distal limb muscles			
BTD	biotinidase deficiency	253260	AR	Symptoms usually appeared by about 3 months of age with seizures as the most frequent initial symptom. Hypotonia, ataxia.	Diffuse cerebral and cerebellar atrophy	hearing loss, optic atrophy, skin rash, alopecia	
C12ORF65	Spastic paraplegia 55, autosomal recessive	615035	AR	reduced upper-extremity strength & dexterity, lower-extremity muscle atrophy, & motor sensory neuropathy		Reduced visual acuity (w/central scotoma & optic atrophy)	
CACNA1A	Developmental and epileptic encephalopathy 42	617106	AD	ID, autism, ataxia, seizures	Cerebellar atrophy	Nystagmus, abnormal eye movements	
CAMK4	NA	NA	AD	Developmental delay, intellectual disability, autism, ataxia, and mixed hyperkinetic movement disorder including severe generalized dystonia			PMID: 30262571
CDKL5	Early infantile epileptic encephalopathy-2	300672	XLD	Severe global developmental delay resulting in ID and poor motor control. Seizures.			
CEP290	Joubert syndrome 5	610188	AR	psychomotor delay, hypotonia, ataxia, oculomotor apraxia, and neonatal breathing abnormalities	'molar tooth sign' consisting of cerebellar vermis hypoplasia or aplasia, thick and maloriented superior cerebellar peduncles, and abnormally deep interpeduncular fossa.		
CLN3	Neuronal ceroid lipofuscinosis-3	204200	AR	seizures and gradual mental and motor dysfunction, myoclonus, parkinsonism, and a severe dysarthria, behavioral problems with angry outbursts, physical violence, and features of depression	Cerebral atrophy	gradual visual loss with macular degeneration, optic atrophy, and/or retinitis pigmentosa.	
COL4A1	Porencephaly 1	175780	AD	ID, seizures, spasticity, dystonia, migraine, hemiparesis	Porencephaly, leukoencephalopathy, schizencephaly, carotid aneurysms, microbleeds, lacunar infartcs, parenchymal hemorrhage	Ophthalmological abnormalities, renal abnormalities, cardiac arrhytmia, muscle cramps, myopathy	
COL4A2	Porencephaly 2	614483	AD	Developmental delay, spasticity, hemiplegia, seizures	Porencephaly, leukoencephalopathy, schizencephaly, parenchymal hemorrhage, carotid aneurysms	Opthalmological abnormalities	

COLGALT1	Brain small vessel disease 3	618360	AR	Developmental delay, spastic quadriplegia, intellectual disability	Cerebral hemorrhage, porencephaly, brain small vessel disease, intracranial calcifications	
CTNNB1	Neurodevelopmental disorder with spastic diplegia and visual defects; NEDSDV	615075	AD	ID, hypotonia, spastic paraplegia, microcephaly	Hypoplasic corpus callosum	Ophthalmological abnormalities, fair skin
CYP2U1	Spastic paraplegia 56, autosomal recessive	615030	AR	ID, spastic paraplegia, dystonia, neuropathy	White matter lesions, thin corpus callosum, calcifications globus pallidus	
CYP2U1	Spastic paraplegia 56, autosomal recessive	615030	AR	Upper-extremity involvement, upper-extremity dystonia, ID, axonal neuropathy	thin corpus callosum, brain white-matter disturbance, basal ganglia calcifications	
CYP7B1	Spastic paraplegia 5A, autosomal recessive	270800	AR	Axonal neuropathy, Distal or generalized muscle atrophy, spasticity	White-matter abnormalities on MRI	
DARS	Hypomyelination with brainstem and spinal cord involvement and leg spasticity	615281	AR	severe lower limb spasticity, nystagmus, hyperreflexia, and extensor plantar responses	extensive white matter abnormalities involving the supratentorial white matter, brainstem, cerebellar peduncles, and dorsal columns and lateral corticospinal tracts of the spinal cord.	
DBT	maple syrup urine disease	248600	AR	progressive neurologic signs of altering hypotonia and hypertonia, reflecting a severe encephalopathy. Seizures and coma usually occur, followed by death if untreated	White matter signal abnormalities, diffusion abnormalities	
DDC	Aromatic L-amino acid decarboxylase deficiency	608643	AR	vegetative symptoms, oculogyric crises, dystonia, and severe neurologic dysfunction, usually beginning in infancy or childhood		Temperature instabilities, sweating
DDHD1	Spastic paraplegia 28, autosomal recessive	609340	AR	early-onset, slowly progressive lower-limb spasticity resulting in walking difficulties. Cerebellar oculomotor disturbance. Axonal neuropathy.		Scoliosis, pes cavus
DDHD2	Spastic paraplegia 54, autosomal recessive	615033	AR	Psychomotor delay, ID, progressive spasticity (leading to foot contractures), Dysarthria, dysphagia, strabismus	thin corpus callosum, periventricular white-matter abnormalities	optic hypoplasia
DGUOK	Mitochondrial DNA depletion syndrome 3	251880	AR	ID, spastic diplegia	Cerebral atrophy	Failure to thrive, hepatomegaly, splenomegaly
DHPR	BH4-deficient hyperphenylalaninemia	261630	AR	progressive encephalopathy combining mental retardation, epilepsy, and pyramidal, cerebellar, and extrapyramidal signs	Intracerebral calcifications	Episodic hyperthermia
DLD	dihydrolipoamide dehydrogenase deficiency	246900	AR	Microcephaly, hypotonia, dystonia, ataxia, seizures, episodic encephalopathy	subacute necrotizing encephalomyelopathy	optic atrophy, metabolic acidosis, hypertrophic cardiomyopathy

DYRK1A	Mental retardation, autosomal dominant 7	614104	AD (DN)	ID, hypotonia, hypertonia, ataxia, abnormal gait, seizures, autistic features	Unspecific	Ophthalmological abnormalities, hearing loss	
EIF2B1	Leukoencephalopathy with vanishing white matter	603896	AR	acute encephalopathy, spastic paraparesis, neurodegeneration, dementia	diffuse cerebral hemispheric leukoencephalopathy		
EIF2B2	Leukoencephalopathy with vanishing white matter	603896	AR	acute encephalopathy, spastic paraparesis, neurodegeneration, dementia	diffuse cerebral hemispheric leukoencephalopathy		
EIF2B3	Leukoencephalopathy with vanishing white matter	603896	AR	acute encephalopathy, spastic paraparesis, neurodegeneration, dementia	diffuse cerebral hemispheric leukoencephalopathy		
EIF2B4	Leukoencephalopathy with vanishing white matter	603896	AR	acute encephalopathy, spastic paraparesis, neurodegeneration, dementia	diffuse cerebral hemispheric leukoencephalopathy		
EIF2B5	Leukoencephalopathy with vanishing white matter	603896	AR	acute encephalopathy, spastic paraparesis, neurodegeneration, dementia	diffuse cerebral hemispheric leukoencephalopathy		
ELP2	Autosomal recessive mental retardation-58	617270	AR	truncal hypotonia, lower limb spasticity with hyperreflexia, choreoathetosis, and stereotypical movements. Mild-severe ID. Agressive behaviour.	Normal		
EPT	Complex spastic paraplegia	NA	AR	Spastic paraplegia, cerebellar ataxia, dysarthria, nystagmus	White matter abnormalities	Mild retinal tortuosity, mild retinal pigment disturbance, short stature, microcephaly, high arched palate, cleft palate, bifid uvula	PMID: 29500230
ERLIN2	Spastic paraplegia 18, autosomal recessive	611225	AR	early-onset spasticity, mental retardation, epilepsy	Thin corpus callosum		
F10	Factor X deficiency	227600	AR	Intracranial hemorrhage		Epistaxis, hemarthrosis, bleeding diathesis	
FAM126A	Hypomyelinating leukodystrophy-5	610532	AR	Truncal hypotonia, pyramidal signs, muscle weakness and wasting of the lower limbs associated with a peripheral motor neuropathy. Cerebellar signs. Mild mental retardation , seizures (in 1 patient).	diffuse cerebral hypomyelination, whereas the cortex and deep gray matter structures were preserved.	congenital cataract	
FAM126A	Leukodystrophy, hypomyelinating, 5	610532	AR	slowly progressive pyramidal and cerebellar dysfunction, muscle weakness and wasting prevailingly in the lower limbs, and most became wheelchair-bound. Mental deficiency ranged from mild to moderate.	diffuse cerebral hypomyelination, whereas the cortex and deep gray matter structures were preserved.	congenital cataract	
FBXO31	ND	ND	AD (DN)	Spastic-diplegic CP, ID, expressive language disorder, ADHD	ventricular dilatation, thin corpus callosum	Strabism, constipation, cleft palate	PMID: 32989326
FH	Fumarase deficiency	606812	AR	Relative macrocephaly, failure to thrive, hypotonia, seizures, developmental delay	Polymicrogyria, angulation of the frontal horns, enlarged ventricles, decreased periventricular white matter, and small brainstem.	Optic nerve hypoplasia or pallor. Metabolic acidosis, necrotizing enterocolitis, liver failure. Frontal	

						bossing, hypertelorism, depressed nasal bridge, anteverted nares, and high-arched palate.
FLVCR2	Fowler syndrome/ Proliferative vasculopathy and hydranencephaly-hydrocephaly syndrome	225790	AR	Joint contractures, severe developental delay, fetal akinesia deformation sequence	Hydranencephaly, diffuse ischemic lesions of the brain stem, basal ganglia, and spinal cord with calcifications	Intrauterine growth retardation, glomeruloid vascular proliferation in the retina
FOXC1	Anterior segment dysgenesis-3	601631	AD	Cerebral small vessel disease	White matter hyperintensities, dilated perivascular spaces, and lacunar infarction	Axenfeld-Rieger anomaly
FOXG1	Congenital variant of Rett syndrome	613454	AD (DN)	severe postnatal microcephaly, severe mental retardation with absent language , deficient social reciprocity resembling autism, combined stereotypies, and dyskinesias, such as dystonia, chorea, and athetosis. The patients also developed epilepsy after age 3 months and showed poor sleep patterns, irritability in infancy	simplified gyral pattern and reduced white matter volume in the frontal lobes, corpus callosum hypogenesis, and variable mild frontal pachygyria	recurrent aspiration, and gastroesophageal reflux.
GAD1	Autosomal recessive spastic cerebral palsy-1	603513	AR	Cerebral palsy, diplegia, quadriplegia, microcephaly, contractures		Scoliosis
GALC	Krabbe disease	245200	AR	extreme irritability, spasticity, and developmental delay. There is severe motor and mental deterioration, leading to decerebration and death. Spastic paraparesis. Neuropathy.	Diffuse cerebral atrophy, demyelination, ventriculomegaly	Optic atrophy, blindness, deafness
GALT	galactosemia	230400	AR	Hypotonia, verbal dyspraxia, ID, motor abnormalities		jaundice, hepatosplenomegaly, hepatocellular insufficiency, food intolerance, hypoglycemia, renal tubular dysfunction, sepsis, and cataract.
GAMT	Cerebral creatine deficiency syndrome 2	612736	AR	developmental delay/regression, mental retardation, severe disturbance of expressive and cognitive speech, intractable seizures and hypotonia, ataxia, extrapyramidal movement disturbances	Myelination delay	
GBA	Gaucher disease type II	230900	AR	rapid progression of neurologic disease with spasticity, rigidity, refractory seizures, and oculomotor abnormalities.	Cerebral atrophy, brain stem degeneration	Squint, oculomotor apraxia, hepatomegaly, splenomegaly, anemia, thrombocytopenia
GCH1	Dystonia, DOPA-responsive, with or without hyperphenylalaninemia	128230	AR/ AD	Dystonia, ataxia, hyperreflexia, parkinsonism, extrapyramidal signs, cerebellar signs		Scoliosis, pes cavus, torticollis

GFAP	Alexander disease	203450	AD	seizures, megalencephaly/ macrocephaly, developmental delay, and spasticity, ataxia, bulbar or pseudobulbar symptoms.	Cerebral white matter abnormalities, preferentially affecting the frontal region. Brain stem atrophy.	
GJA12	Hypomyelinating leukodystrophy-2	608804	AR	Nystagmus, impaired motor development, ataxia, choreoathetotic movements, dysarthria, and progressive spasticity, neuropathy.	Characteristic diffuse T2-weighted hyperintensities consistent with hypomyelination.	Optic atrophy
GJC2	Spastic paraplegia 44, autosomal recessive/ Leukodystrophy, hypomyelinating, 2	613206/ 608804	AR	ID, slowly progressive SPG, dysarthria, & upper-extremity involvement/ early-onset dysmyelinating disorder w/nystagmus, psychomotor delay, progressive spasticity, ataxia	Hypomyelinating leukoencephalopathy	
GLB1	GM1-gangliosidosis	230500	AR	psychomotor deterioration, seizures, dystonia or gait or speech disturbance.		macular cherry-red spots, skeletal dysplasia, hepatosplenomegaly, cardiomyopathy, coarse facial features
GLDC, AMT, GCSH	Glycine encephalopathy/ nonketotic hyperglycinemia	605899	AR	lethargy, hypotonia, and myoclonic jerks, and progressing to apnea, intractable seizures, profound mental retardation, chorea, and vertical gaze palsy		
GLRA1	hyperekplexia-1	149400	AR/ AD	Exaggerated startle response, infantile hypertonia and hypokinesia, nocturnal myoclonus, an easily elicited head retraction reflex, apnea		Umbilical and inguinal hernias
GM2A	GM2-gangliosidosis, AB variant	272750	AR	Abnormal behavior, including increased anxiety and phobias, spastic quadriparesis, limb dystonia, pyramidal signs, and generalized chorea. The hyperkinetic disorder gradually gives way to a rigid akinetic state. Exagerrated sartle response.	Cerebral atrophy,	Cherry-red macular spots
GNAO1	Neurodevelopmental disorder with involuntary movements	617493	AD	Developmental delay, hypotonia, dystonic/ hyperkinetic CP, chorea	Cerebral atrophy, thin corpus callosum	
GNB1	Mental retardation, autosomal dominant 42	616973	AD	ID, hypotonia, spasticity, dyskinesia, ADHD, ASS, seizures	Mostly normal. 1 case polymicrogyria	
GRID2	Spinocerebellar ataxia, autosomal recessive 18		AR/ AD (DN)	nystagmus, hypotonia, delayed psychomotor development, incoordination of gross motor movements, ataxia, dysarthria, nystagmus, pyramidal tract involvement.	Cerebral and cerebellar atrophy	
GRIN1	Neurodevelopmental disorder with or without hyperkinetic movements and	617820	AR/ AD (DN)	Profound developmental delay, ID, hypotonia, spasticity, dyskinesia, chorea, seizures	Thin corpus callosum, cerebral atrophy, plymicrogyria	Feeding difficulties, poor visual contact

	seizures, autosomal recessive/ dominant					
GRIN2B	Mental retardation, autosomal dominant 6	613970	AD (DN)	Developmental delay, ID, seizures, spasticity, dystonia, dyskinesias, choreiform movements	Malformations of cortical development (some patients)	
HESX1	Septooptic dysplasia	182230	AR/ AD	Seizures, developmental delay, and cerebral palsy	absence of the corpus callosum and septum pellucidum	optic nerve hypoplasia, pituitary gland hypoplasia, hypopituitarism
HEXA	Tay-Sachs disease/ GM2-gangliosidosis, several forms	272800	AR	muscle atrophy beginning distally, pes cavus, foot drop, spasticity, mild ataxia of limbs and trunk, dystonia, and dysarthria, dementia, neuropathy	Leukoencephalopathy	Cherry red spot, blindness
HIKESHI	Leukodystrophy, hypomyelinating, 13	616881	AR	infantile onset of delayed psychomotor development, axial hypotonia, and spasticity	delayed myelination and periventricular white matter abnormalities	
HPRT1	Lesch-Nyhan syndrome	300322	XLR	mental retardation, spastic cerebral palsy, choreoathetosis, dystonia, self-destructive biting of fingers and lips		uric acid urinary stones, gout, (megaloblastic) anemia
HSPD1	Leukodystrophy, hypomyelinating, 4	612233	AR	Hypotonia, nystagmus, and psychomotor developmental delay, followed by appearance of prominent spasticity, developmental arrest, and regression.	thin corpus callosum and various degrees of ventricular enlargement, no myelination	
IFIH1	Aicardi-Goutieres syndrome 7	615846	AD	delayed psychomotor development, axial hypotonia, spasticity, microcephaly, spastic-dystonic tetraparesis, and lack of speech.	basal ganglia calcification, cerebral atrophy, and deep white matter abnormalities.	vasculitic rash, lymphadenopathy, serositis with pericardial effusion, and abnormal serum autoantibodies, acute nephrotic syndrome.
ISG15	immunodeficiency-38 with basal ganglia calcifications	616126	AR		Cerebral calcifications	Susceptibility to mycobacterial disease, lymphadenopathy
ITPR1	spinocerebellar ataxia-29	117360	AD	delayed motor development and mild cognitive delay, gait and limb ataxia, nystagmus, dysarthria, and tremor	Cerebellar (vermis) atrophy	
JAM3	Hemorrhagic destruction of the brain, subependymal calcification, and cataracts	613730	AR	Profound developmental delay, spasticity, seizures	Intraparenchymal brain hemorrhage, ventriculomegaly, porencephaly, subependymal calcifications	
KANK1/ ANKRD15	Cerebral palsy, spastic quadriplegic, 2	612900	(deletions inherited from unaffected fathers)	Congenital hypotonia, spastic quadriplegia, nystagmus, mental retardation.	brain atrophy and ventriculomegaly.	

KCNC3	Spinocerebellar ataxia 13	605259	AD	childhood-onset cerebellar gait ataxia associated with cerebellar dysarthria, moderate mental retardation, and mild developmental delay in motor acquisition. Nystagmus and pyramidal signs	Moderate cerebellar and pontine atrophy	
KCNQ2	Epileptic encephalopathy, early infantile, 7	613720	AD	ID, dystonic CP, spastic quadriparesis	Hyperintensities in the basal ganglia and/or thalamus, thin corpus callosum, reduced posterior white matter volume	
KDM5C	Mental retardation, X-linked, syndromic, Claes-Jensen type	300534	XLR	evere mental retardation, slowly progressive spastic paraplegia, facial hypotonia, aggressive behavior and strabismus, seizures, microcephaly		Short stature, dysmorphisms
KIDINS220	Spastic paraplegia, intellectual disability, nystagmus, and obesity	617296	AD (nonsense de novo that escape NMD)/ AR	ID, spastic paraplegia, macrocephaly	ventricular dilatation, cerebral atrophy, delayed myelination	Obesity, nystagmus
KIF1A	Spastic paraplegia 30, autosomal recessive	610357	AR	unsteady spastic gait and hyperreflexia of the lower limbs. Mildly impaired sensation and cerebellar involvement. Axonal sensorimotor peripheral neuropathy. Normal cognition.	Mild cerebellar atrophy	
KIF1C	Spastic ataxia 2, autosomal recessive	611302	AR	Spasticity, ataxia	Normal	
KIF5A	Spastic paraplegia 10, autosomal dominant	604187	AD	lower limb spasticity, hyperreflexia, and weakness, leading to impaired gait, mild upper limb hyperreflexia, pes cavus, axonal sensorimotor peripheral neuropathy, distal sensory impairment.		
L1CAM	Hydrocephalus due to aqueductal stenosis/ MASA syndrome	307000	XLR	mental retardation, and often includes spastic paraparesis and adducted thumbs	Hydrocephalus, aqueductal stenosis, agenesis of corpus callosum	Hypoplasia and contracture of the thumb
L1CAM	MASA syndrome	303350	XLR	Hydrocephalus, aphasia, & adducted thumbs variably present		
L2HGDH	L-2-hydroxyglutaric aciduria	236792	AR	Macrocephaly, spastic tetraparesis, dystonia, extrapyramidal signs, seizures, regression	subcortical leukoencephalopathy, cerebellar atrophy, and signal changes in the putamina and dentate nuclei, demyelinated with cystic cavities	hearing loss, optic atrophy, strabism
LAMA2	Merosin-deficient congenital muscular dystrophy type 1A	607855	AR	hypotonia, poor suck and cry, and delayed motor development, areflexia. Mental retardation and/or seizures occur only rarely	Periventricular white matter abnormalities, abnormal cortical gyration (polymicrogyria)	

LIS1	lissencephaly-1 / Subcortical laminar heterotopia	607432	AD (DN)	Hypotonia, spastic quadripareses, seizures, severe developmental delay	Agyria, pachygyria, sybcortical band heterotopia, Corpus callosum abnormalities, cerebellar hypoplasia, brainstem hypoplasia, enlarged ventricles, White matter abnormalities, prominent perivascular spaces.	
MARS2	Spastic ataxia 3, autosomal recessive	611390	AR	spastic ataxia and brain white matter changes	Cerebral and cerebellar atrophy	
MCPH1	Primary microcephaly-1	251200	AR	Seizures, developmental delay, microcephaly		Short stature
MECP2	Rett syndrome/Encephalopathy, neonatal severe	312750/ 300673	XLD/ XLR	deterioration progressed to severe dementia, autism, loss of purposeful use of the hands, jerky truncal ataxia, and 'acquired' microcephaly, mainly spastic paraparesis, vasomotor disturbances of the lower limbs, and epilepsy	Cortical atrophy (frontal area)	Hyperventilation, apnea, scoliosis, cold feet
MOCS1/ MOCS2/ GPHN	Molybdenum cofactor deficiency	252150	AR	seizures (72%), feeding difficulties (25%) and hypotonia (11%), developmental delay (9%), hemiplegia (2%), lens dislocation (2%), and hyperreflexia	brain atrophy, neuronal loss, astrocytic gliosis, cystic changes in the subcortical white matter, thin corpus callosum, enlarged ventricles, and demyelination	dysmorphic facial features, including long face with puffy cheeks, widely spaced eyes, elongated palpebral fissures, thick lips, long philtrum, and small nose.
MRPS25	Combined oxidative phosphorylation deficiency 50	619025	AR	Dyskinetic cerebral palsy, myopathy, developmental delay	Partial agenesis of corpus callosum	Short stature, adrenal insufficiency
MTHFR	Homocystinuria due to MTHFR deficiency	236250	AR	poor sucking, hypotonia, microcephaly, and lethargy in the newborn period, microcephaly, fits of apnea, seizures, and coma, gait abnormalities, psychatric illness	Cerebral atrophy, abnormal gyral pattern, diffuse white matter hyperintensities, hypoplasia of the cerebellum and brainstem, and thinning of the corpus callosum	
MTOR	Smith-Kingsmore syndrome	616638	AD	Macrocephaly, ID, seizures, hypotonia	Mild prominence of the ventricular system, corpus callosum hypoplasia, generalized white matter loss, small mesencephalon, pons and medulla, perisylvian polymicrogyria, heterotopic gray matter in the right frontal lobe	Facial dysmorphisms, curly hair, cafe-au-laitspots, macrosomia, rhizomelic limb shortening, thrombocytopenia
MTPAP	Spastic ataxia 4, autosomal recessive	613672	AR	early childhood onset of a slowly progressive neurodegenerative disorder characterized primarily by cerebellar ataxia, spastic paraparesis, dysarthria, and optic atrophy		
MTTP	Abetalipoproteinemia	200100	AR	Neuropathy, ataxia	Demyelination	retinal degeneration, coagulopathy, hepatic steatosis. Acanthocytosis
MUT	Methylmalonic aciduria, mut(0) type	251000	AR	lethargy, hypotonia, developmental delay, coma, strokes	Basal ganglia ischemic stroke, cerebellar hemorrhage, globus	Failure to thrive, recurrent vomiting, dehydration, respiratory distress,

					pallidus involvement, myelination delay	hepatomegaly, renal insufficiency, chronic pancreatitis, anemia, and optic atrophy, cardiomyopathy.	
NIPA1	Spastic paraplegia 6, autosomal dominant	600363	AD	hyperreflexia and spasticity in the lower limbs, weakness of hip flexion and ankle dorsiflexion, extensor plantar responses, diminished vibratory sense in the feet, and pes cavus. Muscle atrophy. Seizures, postural tremor.		Mild bladder disturbances	
NKX2.1	Choreoathetosis, hypothyroidism, and neonatal respiratory distress/ Chorea, hereditary benign	610978	AD	Movement abnormalities begin with muscular hypotonia followed by the development of chorea, athetosis, dystonia, ataxia, and dysarthria. Developmental delay in some.		neonatal respiratory distress. Hypothyroidism, congenital. Hypoplasia of the thyroid gland in some patients	
NKX6.2	Spastic ataxia 8, autosomal recessive, with hypomyelinating leukodystrophy	617560	AR	Delayed motor development, neonatal hypotonia, cerebellar ataxia, pyramidal tract signs, dystonia, peripheral neuropathy	Cerebellar atrophy and hypomyelinating leukodystrophy		
NOTCH3	recessive early-onset arteriopathy and cavitating leukoencephalopathy	NA	AR (homozygous null-mutation)	childhood-onset arteriopathy	Cavitating leukoencephalopathy with cerebral white matter abnormalities presented as diffuse cavitations, multiple lacunar infarctions and disseminated microbleeds.		PMID: 25870235
NOTCH3	CADASIL	125310	AD	Strokes, dementia, seizures, migraine, gait disturbances, psychiatric disturbances	Lacunar infarcts, microbleeds, leukoencephalopathy, subcortical lacunar lesions		
NPC1	Niemann Pick C		AR	neurologic abnormalities: ataxia, grand mal seizures, and loss of previously learned speech. Spasticity is striking and seizures, particularly myoclonic jerks, are common. Dystonia, vertical supranuclear gaze palsy, dementia, and psychiatric manifestations.	Neuronal loss, particularly of cerebellar Purkinje cells	Hepatosplenomegaly, cholestasis. Foamy Niemann-Pick cells and 'sea-blue' histiocytes with distinctive histochemical and ultrastructural appearances are found in the bone marrow.	
NPC2	Niemann Pick C	607625	AR	vertical supranuclear gaze paresis, dysarthria, and cognitive decline , expressive aphasia, ataxia and athetoid movements, facial dyskinesias and bradykinesia.		Respiratory failure, Foam cells on bone marrow biopsy<br>- 'Sea-blue' histiocytes	
NPHP1	Joubert	609583	AR	gross motor delay, mild cognitive impairment, hypotonia, congenital head tilt, abnormal eye movements consistent with oculomotor apraxia,	hypoplasia of the cerebellar vermis and the 'molar tooth sign	nephronophthysis	
NT5C2	Spastic paraplegia 45, autosomal recessive	613162	AR	Spastic paraplegia, ID, ASS, ADHD	White matter abnormalities, thin/ dysplastic corpus callosum	Pes equinovarus	
PAK3	Mental retardation, X-linked 30/47	300558	XLR	microcephaly, marked hypotonia, and oromotor dysfunction with drooling and speech			

				difficulties. Behavioral features, including aggression, hyperactivity, and agitation. Epilepsy		
PANK2	Neurodegeneration with brain iron accumulation 1	234200	AR	Developmental delay, extrapyramidal syndrme, spasticity, cognitive decline	Decreased signal intensity in pallidal nuclei with central hyperintensity, brain atrophy	Ophthalmological abnormalities, feeding difficulties
PCCA	Propionicacidemia	606054	AR	lethargy and ketosis, developmental retardation	Cerebral atrophy, cerebellar hemorrhage	hyperglycinemia, ketoacidosis, neutropenia, and thrombocytopenia, episodic vomiting, intolerance to protein.
PCCB	Propionicacidemia	606054	AR	lethargy and ketosis, developmental retardation	Cerebral atrophy, cerebellar hemorrhage	hyperglycinemia, ketoacidosis, neutropenia, and thrombocytopenia, episodic vomiting, intolerance to protein.
PCYT2	Spastic paraplegia 82, autosomal recessive	618770	AR	Spastic paraplegia, cerebellar ataxia, dysarthria, nystagmus	Progressive cortical atrophy, thinning of cc and white matter abnormalities	Mild growth retardation, growth hormone deficiëncy
PDHA1	Pyruvate dehydrogenase E1-alpha deficiency	312170	XLD	Hypotonia, lethargy, seizures, mental retardation, spasticity, ataxia, choreoathetosis	Cerebral atrophy, Cystic lesions in the basal ganglia, brainstem, and cerebral hemispheres similar to Leigh syndrome (256000), agenesis of corpus callosum, ventricular dilatation	Minimal metabolic abnormalities possible
PDHX	Lacticacidemia due to PDX1 deficiency	245349	AR	Seizures, static cerebral palsy-like encephalopathy with spasticity and severe to profound mental retardation		Lactic acidosis crisis in first days/ months
PGN	pastic paraplegia-7	607259	AR (sporadically AD)	lower limb spasticity, pyramidal signs, lower limb hyperreflexia, supranuclear palsy, nystagmus, and cerebellar dysarthria	Cortical and cerebellar atrophy	Optic atrophy, nystagmus, scoliosis
PHYH	Refsum disease	266500	AR	retinitis pigmentosa, peripheral neuropathy, cerebellar ataxia, and elevated protein levels in the cerebrospinal fluid		Cardiomyopathy, ichthyosis, hearing loss
PIK3CA	Megalencephaly-capillary malformation-polymicrogyria syndrome	602501	AD-DN/ mosaicism	primary megalencephaly, ID	Cortical malformations, polymicrogyria, ventriculomegay, brain asymmetry	Overgrowth, asymmetry, cutaneous vascular lesions, syndactyly, polydactyly, connective tissue dysplasia
PLA2G6	Infantile neuroaxonal dystrophy 1	256600	AR	hypotonia, severe developmental delay with mental retardation, motor regression, and no eye contact. Tetraparesis, extrapyramidal symptoms, ataxia	Cerebral atrophy, cerebellar atrophy with signal hyperintensity in the cerebellar cortex seen on T2-weighted MRI, increased iron deposition in the basal ganglia	

PLAA	Neurodevelopmental disorder with progressive microcephaly, spasticity, and brain anomalies	617527	AR	infantile onset of progressive microcephaly and spasticity and severe global developmental delay resulting in profound mental retardation and severely impaired or absent motor function.	myelinating abnormalities and white matter lesions consistent with a leukoencephalopathy, as well as structural anomalies, including thin corpus callosum, gyral abnormalities, and cerebral or cerebellar atrophy	
PLP1	Pelizaeus-Merzbacher disease	312080	XLR	nystagmoid eye movement and jerking, rolling head movements or head tremor. Nystagmus disappears and ataxia, spasticity, and involuntary movements become manifest, as well as microcephaly	Dysmyelination, abnormal white matter signals	Optic atrophy
PMM2	congenital disorder of glycosylation type Ia	212065	AR	axial hypotonia, abnormal eye movement, psychomotor retardation, peripheral neuropathy, ataxia, seizures, stroke-like episodes	Olivopontocerebellar hypoplasia	retinitis pigmentosa, abnormal distribution of subcutaneous fat, nipple retraction, hypogonadism, infections, liver insufficiency, cardiomyopathy
PNP	Immunodeficiency due to purine nucleoside phosphorylase deficiency	613179	AR	Spastic diplegia, tetrapareses, PMR, ataxia, tremor, hypotonia, hypertonia	Cerebral vasculitis	Immunodeficiency
POLG1	Mitochondrial DNA depletion syndrome 4A (Alpers type)	203700	AR	psychomotor retardation, intractable epilepsy, hypotonia, hypertonia, ataxia, dementia	Progressive brain atrophy, cerebellar atrophy, gliosis	Liver failure
POLR1C	Leukodystrophy, hypomyelinating, 11	616494	AR	delayed psychomotor development, loss or lack of independent ambulation, abnormal cognition, tremor, ataxia, spasticity, and cerebellar findings	hypomyelination and thin corpus callosum	hypogonadotropic hypogonadism
POLR3A	Leukodystrophy, hypomyelinating, 7, with or without oligodontia and/or hypogonadotropic hypogonadism	607694	AR	neurodegenerative disorder , childhood onset of progressive motor decline manifest as spasticity, ataxia, tremor, and cerebellar signs, as well as mild cognitive regression.	Leukodystrophy, hypomyelination, thinning of corpus callosum, cortical and cerebellar atrophy	hypodontia or oligodontia and hypogonadotropic hypogonadism
POLR3B	Leukodystrophy, hypomyelinating, 8, with or without oligodontia and/or hypogonadotropic hypogonadism	614381	AR	early childhood onset of cerebellar ataxia and mild intellectual disabilities	high-intensity areas in the white matter on T2-weighted images, consistent with diffuse cerebral hypomyelination, as well as cerebellar atrophy, and hypoplastic corpus callosum.	hypogonadotropic hypogonadism
PPT1 (CLN1)	neuronal ceroid lipofuscinosis-1	256730	AR	Mental retardation, loss of speech, minor motor seizures, regression of motor development, and ataxia.	Cerebral atrophy, progressive, hypointensities of the thalami, high signal of the white matter later on MRI	optic atrophy and macular and retinal changes
PYCR2	Leukodystrophy, hypomyelinating, 10	616420	AR	progressive microcephaly, severely delayed psychomotor development, truncal hypotonia, appendicular hypertonia, hyperreflexia, and severe muscle wasting	hypomyelination, decreased white matter volume, and thin corpus callosum and brainstem	

RARS	Leukodystrophy, hypomyelinating, 9	616140	AR	delayed psychomotor development, spasticity, and nystagmus in the first year of life. Additional neurologic features such as ataxia and abnormal movements may also occur	diffuse hypomyelination affecting all regions of the brain		
REEP1	Spastic paraplegia 31, autosomal dominant	610250	AD	spastic paraplegia mainly characterized by proximal weakness of the lower extremities with brisk reflexes and spastic gait abnormalities, onset in childhood possible		Urinary urgency	
RHOB	ND	ND	AD (DN)	Spastic-dystonic CP			PMID: 32989326
RNASEH2A	Aicardi-Goutieres syndrome 4	606034	AR	Developmental delay, spasticity, seizures	Ventricular dilatation, leukodystrophy, cerebral atrophy, brain stem atrophy, calcifications	Hepatomegaly, splenomegaly, poor feeding	
RNASEH2B	Aicardi-Goutieres syndrome-2	610181	AR	Neurological dysfunction, progressive, spastic paraplegia, encephalopathy, dystonia	Intracranial calcifications		
RNASEH2C	Aicardi-Goutieres syndrome 3	610329	AR	Developmental delay, hyperreflexia, encephalopathy	Delayed myelination, white matter abnormalities, thin corpus callosum, intracranial calcifications	Chilblains, ophthalmlogical findings	
RNASET2	cystic leukoencephalopathy without megalencephaly	612951	AR	Seizures, spasticity, athetosis, ID, ataxia, dystonia	Brain MRI showed extensive cysts within the anterior temporal lobes, ventricular enlargement, and white matter disease, cerebral calcifications	Hearing loss, nystagmus	
RTN2	spastic paraplegia-12	604805	AD	Lower limb spasticity and hyperreflexia, early onset (1e decade)		Urinary incontinence	
SACS	Spastic ataxia, Charlevoix-Saguenay type	270550	AR	cerebellar ataxia, pyramidal tract signs, and peripheral neuropathy	Cerebellar vermis atrophy	Swan-neck deformity of the fingers	
SAMHD1	Aicardi-Goutieres syndrome-5	612952	AR	Developmental delay, hypotonia, spasticity	Leukoencephalopathy, intracerebral calcifications, cerebrovascular abnormalities	Chilblains, skin problems, feeding difficulties, mouth ulcers	
SCN8A	infantile epileptic encephalopathy-13	614558	AD	ID, seizures, regression	Cerebral atrophy		
SEPSECS	Pontocerebellar hypoplasia type 2D	613811	AR	ID, seizures, spastic quadriplegia, progressive	Cerebral atrophy, cerebellar atrophy, white matter abnormalities, thin corpus callosum		
SHH	holoprosencephaly-3	142945	AD	Developmental delay, ID, motor disorder	Holoprosencephaly, dilated ventricles	Microcephaly, facial dysmorphisms, cleft lip/ palate, ocular abnormalities	
SIX3	Holoprosencephaly 2	157170	AD	Developental delay, ID, seizures, hypotonia	Holoprosencephaly, corpus callosum agenesis, cerebellar hypoplasia	Microcephaly, facial dysmorphisms, cleft lip/ palate, ocular abnormalities	
SLC16A2	Allan-Herndon-Dudley syndrome	300523	XLD	Severe mental retardation, dysarthria, ataxia, athetoid movements, muscle hypoplasia, and spastic paraplegia with	Delayed myelination, leukodystrophy, improving with age	Nystagmus; contractures, pectus excavatum, thyroid abnormalities	

				hyperreflexia, clonus, and Babinski reflexes, quadriplegia		
SLC17A5	Salla disease	604369	AR	hypotonia, cerebellar ataxia, and mental retardation, athetosis, spasticity	Progressive cerebellar atrophy and dysmyelination	Growth retardation, nystagmus
SLC19A3	Thiamine metabolism dysfunction syndrome-2	607483	AR	Subacute onset, dystonia, paraparesis, ataxia, seizures, and pyramidal signs	Basal ganglia lesions seen on MRI, cortical and subcortical hyperintensities	
SLC2A1	GLUT1 deficiency syndrome	612126/ 606777	AD	ID, paroxysmal exercise-induced dyskinesia, dystonia, choreoathetosis, seizures	Cerebral atrophy	Hypoglycorrhachia (low glucose in CSF), hemolysis
SLC5A6	Publication Subramanian et al Hum Genet 2017		AR	Spastic cerebral palsy, developmental delay, microcephaly	Brain atrophy, thin corpus callosum, wide ventricles	Osteoporosis, fractures, immune deficiency, FTT
SLC6A1	myoclonic-atonic epilepsy	616421	AD	Developmental deley, myoclonic-atonic epilepsy, ID, autism, tremor, ataxia		Scoliosis
SLC6A5	hyperekplexia-3	614618	AR	neonatal hypertonia, an exaggerated startle response to tactile or acoustic stimuli, and life-threatening neonatal apnea episodes.		
SPAST	autosomal dominant spastic paraplegia-4	182601	AD	Spastic paraplegia, pyramidal signs, cognitive decline, ID		Urinary incontinence
SPG11	autosomal recessive spastic paraplegia-11	604360	AR	lower limb spasticity, slight ataxia, and mild sensory impairment, urinary urge incontinence and slow cognitive decline. Axonal loss and demyelinating sensory neuropathy	thin corpus callosum, cerebral atrophy, white matter lesions	
SPG11		604360	AR	ID, upper-extremity weakness, dysarthria, & nystagmus	Thin corpus callosum	
SPR	dopa-responsive dystonia	612716	AR	progressive psychomotor retardation, dystonia, spasticity, ataxia, choreoathetosis. Microcephaly.		Oculomotor apraxia, oculogyric crisis, growth retardation
SPTBN2	spinocerebellar ataxia-5/ autosomal recessive spinocerebellar ataxia-14	600224/615386	AD/AR	PMR, hypotonia, ataxic cerebral palsy, pyramidal signs	Cerebellar atrophy	Nystagmus
ST3GAL5	Salt and pepper developmental regression syndrome	609056	AR	Intellectual disability, choreoathetosis, epilepsy, neurological regression	Cerebral atrophy	Deafness, vision impairment, and skin findings.
STAT2	Pseudo-TORCH type 3	618886	AR	Developmental delay, regression	Intracranial calcifications, white matter abnormalities, cerebral or cerebellar atrophy	Multiorgan dysfunction, poor overall growth
STXBP1	Epileptic encephalopathy, early infantile, 4	612164	AD	ID, hypotonia, tremor, spastic di-/ quadriparesis, seizures	normal	
SUOX	Sulfite oxidase deficiency	606887	AR	Hemiplegia, hypertonia, dystonia, ataxia, choreoathetosis		Ectopia lentis, delayed teething, exzema
SURF1	Leigh syndrome	256000	AR	ID, hypertonia, spasticity, ataxia, seizures	Lesions in basal ganglia, brainstem, cerebellum, thalamus, spinal cord characterized by demyelination, necrosis, gliosis, spongiosis, and capillary proliferation	Optic atrophy, nystagmus, ptosis FTT, respiratory failure

TAF2	Mental retardation, autosomal recessive 40	615599	AR	ID, microcephaly, spasticity, hyperreflexia	Corpus callosum abnormalities, delayed myelination	Nystagmus, pigmentary retinal changes
TECPR2	Spastic paraplegia 49, autosomal recessive	615031	AR	ID, developmental delay, spastic paraparesis	Thin cormus callosum, cerebral atrophy	Recurrent apneic episodes, gastroesophageal reflux
TECPR2	Spastic paraplegia 49, autosomal recessive	615031	AR	Hypotonia, developmental delay, severe ID, Spastic, ataxic, & rigid gait developed in childhood. Epilepsy	Thin corpus callosum & cerebellar atrophy	gastroesophageal reflux, recurrent apneic episodes, mild dysmorphic features
TGIF1	holoprosencephaly-4	142946	AD	Developmental delay, motor problems	Semilobar holoprosencephaly	Cleft lip/ palate
TH	Segawa syndrome, tyrosin hydroxylase deficiency	605407	AR	Ataxia, dystonia, rigidity, involuntary movements		Masked facies, ptosis, oculogyric crises
TPP1 (CLN2)	neuronal ceroid lipofuscinosis-2	204500	AR	refractory epilepsy, mental regression and deterioration, ataxia, myoclonus, and visual loss.	Cerebral atrophy	retinal degeneration
TRAPPC12	Encephalopathy, progressive, early-onset, with brain atrophy and spasticity	617669	AR	small developmental gains in infancy, regression and had severe global developmental deficits. Hypssarthytmia. Truncal hypotonia, appendicular spasticity, dystonia and/or myoclonus, optic atrophy or cortical visual impairment, scoliosis, and dysphagia	pontine hypoplasia, partial agenesis of the corpus callosum, simplified frontal gyri, and diffuse cortical atrophy with enlarged ventricles and relative sparing of the cerebellum	
TREX1	Aicardi-Goutieres syndrome-1	225750	AD/ AR	Developmental delay, ID, spastic tetraparesis, dystonia, seizures, truncal hypotonia	Intracerebral calfications, cerebral atrophy, leukoencephalopathy	Hepatomegaly, splenomegaly, chilblains, acrocyansis of feet
TSEN15	Pontocerebellar hypoplasia, type 2F	617026	AR	cognitive and motor delay, poor or absent speech, seizures, and spasticity	hypoplasia of the cerebellum and pons	
TSEN2	Pontocerebellar hypoplasia type 2B	612389	AR	No psychomotor development, spasticity, dystonia, dyskinesia, axial hypotonia, opisthotonus, seizures	Cerebellar atrophy, brainstem hypoplasia, pontine atrphy, ventricular dilatation, thin corpus callosum	Central visual impairment, feeding difficulties, microcephaly
TSEN54	pontocerebellar hypoplasia type 2A	277470	AR	ID, spasticity, dyskinesia, seizures	Pontine hypoplasia, cerebellar hypoplasia (predominantly hemispheres), dragonfly-like pattern	Central visual impairment
TTPA	Ataxia with isolated vitamin E deficiency	277460	AR	Ataxia, areflexia, early onset		
TUBA1A	lissencephaly-3	611603	AD	ID, spastic tetraplegia, ataxia, seizures	Pachygyria, PMG, thin cc, ventricular dilatation	
TUBA8	Cortical dysplasia, complex, with other brain malformations 8	613180	AR	Profound developmental delay, seizures, hypotonia	Thickened cortex, polymicrogyria, corpus callosum agenesis, colpocephaly, hypoplastic brain stem	
TUBB/ TUBB5	Cortical dysplasia, complex, with other brain malformations 6	615771	AD (DN)	Severe developmental delay, ataxia	Dysmorphic basal ganglia, corpus callosum abnormalities, White matter streaks, brainstem hypoplasia, cerebellar hypoplasia	ophthalmological findings

TUBB2A	Cortical dysplasia, complex, with other brain malformations 5	615763	AD (DN)	Developmental delay, ID seizures, hypotonia	Thin corpus callosum, enlarged ventricles, basal ganglia abnormalities	
TUBB2B	Cortical dysplasia, complex, with other brain malformations 7	610031	AD (DN)	Developmental delay, ID, seizures, spasticity	Malformations of cortical development, cerebellar hypopasia, corpus callosum abnormalities	
TUBB3	Cortical dysplasia, complex, with other brain malformations 1	614039	AD (DN)	Developmental delay, ID, axial hypotonia, spasticity, seizures	Malformations of cortical development, dysmorphic basal ganglia, dysplastic cerebellar vermis, corpus callosum abnormalities	Strabism, nystagmus
TUBB4A	Leukodystrophy, hypomyelinating, 6	612438	AD (DN)	delayed motor development and gait instability, followed by extrapyramidal movement disorders, such as dystonia, choreoathetosis, rigidity, opisthotonus, and oculogyric crises, progressive spastic tetraplegia, ataxia, and, more rarely, seizures.	combination of hypomyelination, cerebellar atrophy, and atrophy or disappearance of the putamen	
TUBG1	Cortical dysplasia, complex, with other brain malformations 4	615412	AD (DN)	Developental delay, spastic tetraplegia, seizures	Agyria, pachygyria, dysmorphic corpus callosum, subcortical band heterotopia	Cataract
UBE3A	Angelman syndrome	105830	AD	Develpmental delay, ID, wide-based gait, hyperreflexia, hypotonia, ataxia, seizures	Mild cortical atrophy	Feeding difficulties, facial dysmorphisms, strabism
USP18	Pseudo-Torch syndrome 2	617397	AR	Hypotonia, seizures, lethargy	Enlarged ventricles, cortical destruction, cerebellar hypplasia, abnormal neuronal migration, cerebral calcification	Hepatomegaly, thrombocytopenia, respiratory insufficiency, extracranial calcifications, infantile demise
VPS11	Leukodystrophy, hypomyelinating, 12	616683	AR	severe global developmental delay apparent since infancy, poor speech or absence of speech, central hypotonia, microcephaly, and poor overall growth. Additional features included seizures, limb hypertonia, joint contractures	enlarged ventricles, decreased periventricular white matter, hypoplastic corpus callosum, and signal abnormalities of the supratentorial white matter.	hearing loss, central visual impairment, optic atrophy.
VPS37A	Spastic paraplegia 53, autosomal recessive	614898	AR	developmental delay, progressive lower-extremity spasticity, & subsequently progressive upper-extremity involvement, mild-to-moderate ID		skeletal dysmorphism (kyphosis & pectus carinatum, variable hypertrichosis
VPS53	Pontocerebellar hypoplasia, type 2E	615851	AR	Profound developmental delay, ID, seizures, spastic quadriplegia, ophistotonus	Cerebral atrophy, cerebellar atrophy, thin corpus callosum	Short stature, microcephaly, optic atrophy, osteoporosis, distal limb edema
YY1AP1	Grange syndrome	602531	AR	Mild-moderate intellectual disability	Thalamic hemorrhage infantile, cerebral hemorrhage, ischemic cerebrovascular disease	Renovascular hypertension, bone fragility
ZC4H2	Wieacker-Wolff syndrome	314580, 301041	XLR/D	ID, hypotonia, spasticity, arthrogryposis multiplex congenita, seizures		Short stature, narrow thorax, facial dysmorphisms

ZFYVE26	spastic paraplegia-15	270700	AR	ID, spasticity, ataxia, neuropathy	Thin corpus callosum, white matter abnormalities, cerebral atrophy	hearing deficit, retinal abnormalities,
ZIC2	holoprosencephaly-5	609637	AD	ID, motor problems	Hydrocephalus, holoprosencephaly	Facial dysmorphisms, cleft lip/ palate
ZNF238= ZBTB18	Mental retardation, autosomal dominant 22	612337	AD	ID, spastic diplegia, microcephaly, seizures	Corpus callosum abnormalities	

Supplementary Table 1. Overview of genes associated with cerebral palsy or cerebral palsy mimics, their associated condition, inheritance pattern and clinical characteristics. AD, autosomal dominant; AR, autosomal recessive; XLR, X-linked recessive; XLD, X-linked dominant; DN, *de novo*; ID, intellectual disability; FTT, failure to thrive; CP, cerebral palsy; SLE, systemic lupus erythematosus.
